# Supplementary material for: Deoxyxylulose 5-Phosphate Synthase Does Not Play a Major Role in Regulating the Methylerythritol 4-Phosphate Pathway in Poplar
Source: Int J Mol Sci. 2024 Apr 10;25(8):4181. doi: 10.3390/ijms25084181 (PMC11049974; doi:10.3390/ijms25084181)
Supplement: Supplementary file 1 [file ijms-25-04181-s001.zip › ijms-2905105-supplementary.pdf]

International Journal of
Molecular Sciences

MDPI

SUPPLEMENTAL DATA

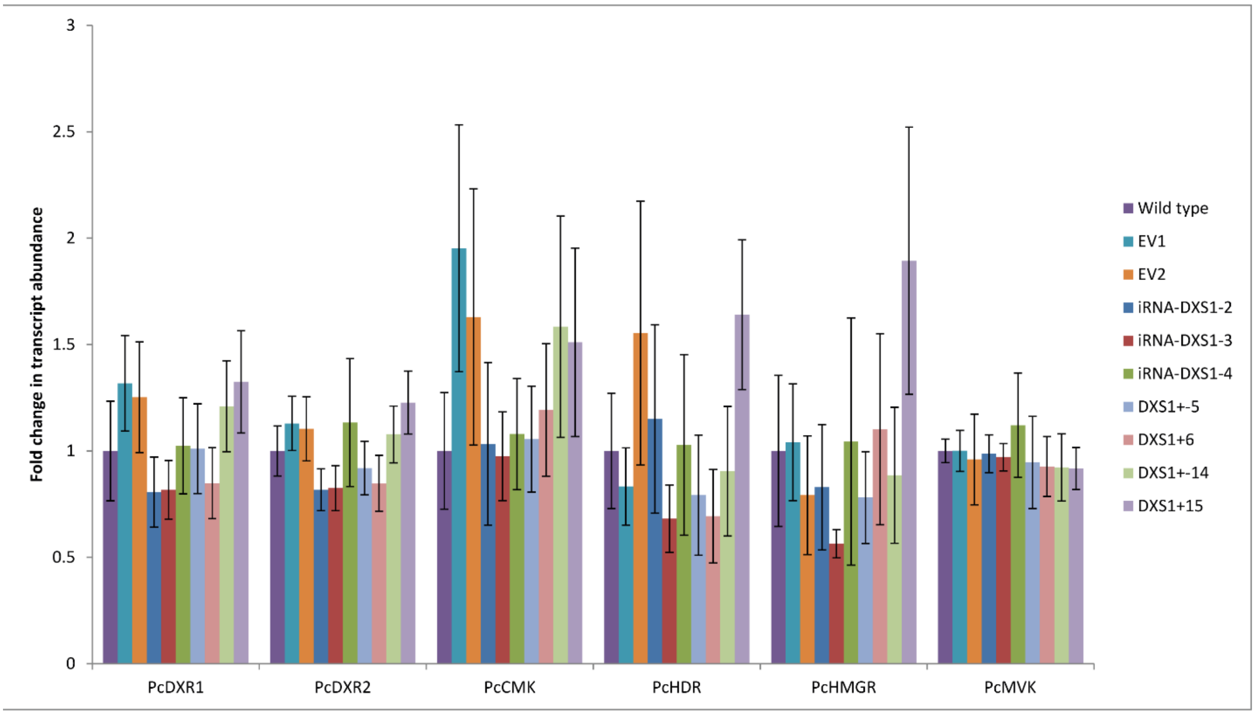

Supplemental Figure S1. Transcript levels of MEP pathway genes (*PcDXR1*, *PcDXR2*, *PcCMK*, *PcHDR*) and MVA pathway genes (*PcHMGR*, *PcMVK*) at midday in transgenic *DXS*-silenced (iRNA-2, iRNA-3, iRNA-4), and overexpression (DXS1+-5, DXS1+-6, DXS1+-14, DXS1+-15) lines compared to wild-type and empty vector (EV1, EV2) controls, silenced (iRNA-2, iRNA-3, iRNA-4), and transgenic overexpression (DXS1+-5, DXS1+-6, DXS1+-14, DXS1+-15)lines. Leaves were subjected to 250 µmol m^{-2} s^{-1} of incident PPFD, 21°C leaf temperature and 380 µmol mol^{-1} of CO_2 for 50 min before taking samples. Relative quantification was performed according to the efficiency corrected model (Pfaffl, 2001). Efficiencies were obtained from the slope of dilution curves using control cDNA diluted from 1 to 1024 times at 4x intervals. Target gene expression was normalized to the *PcActin2* and fold-change values for each gene were calculated by comparison with the mean expression of the same gene in wild-type control plants (dark purple). Error bars indicate the SE of three biological replicates (n=3) analyzed in triplicate SYBR green assays. For abbreviations of genes, see Figure 1 and *HMGR*, 3-hydroxyl-3-methylglutaryl-CoA reductase; *MVK*, mevalonate kinase.

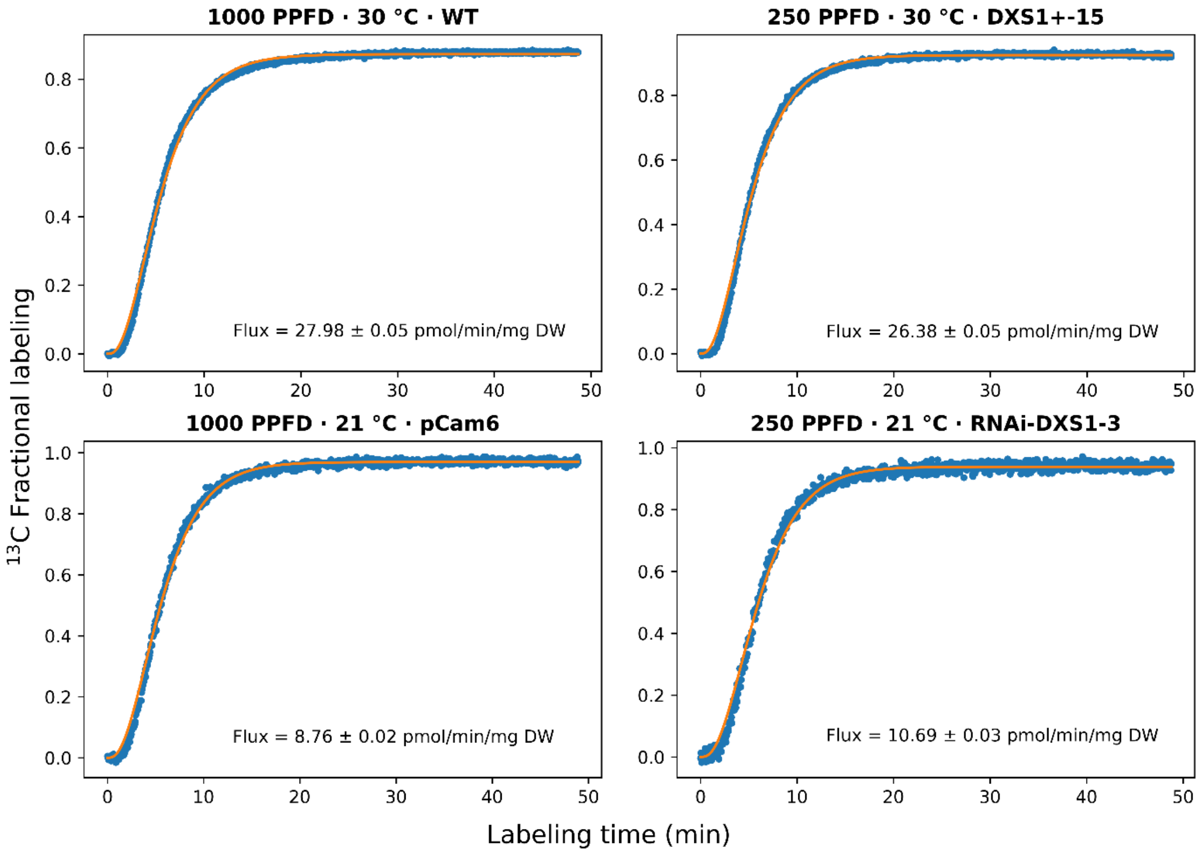

Supplemental Figure S2. Time-courses of the fractional ^{13}C-labeling of the molecular ion of isoprene after incorporation of $^{13}CO_2$ under different environmental conditions of light (1000 or 250 PPFD) and temperature (30 or 21 ^{0}C). Shown are representative examples for each of the wild type (WT), empty vector control (pCam), *DXS1* overexpressing (DXS1+), and *DXS*-silenced (RNAi-DXS1) lines. The fractional labeling was calculated by summing all ^{13}C atoms incorporated into the various isotopologues (see Figure 3) as described under Methods. The experimental data (blue circles) were fitted (orange lines) to Equation (1) as described under Methods after entering the plastidial pool sizes of DXP, MEcDP and IDP+DMADP to calculate the flux as indicated on each graph.

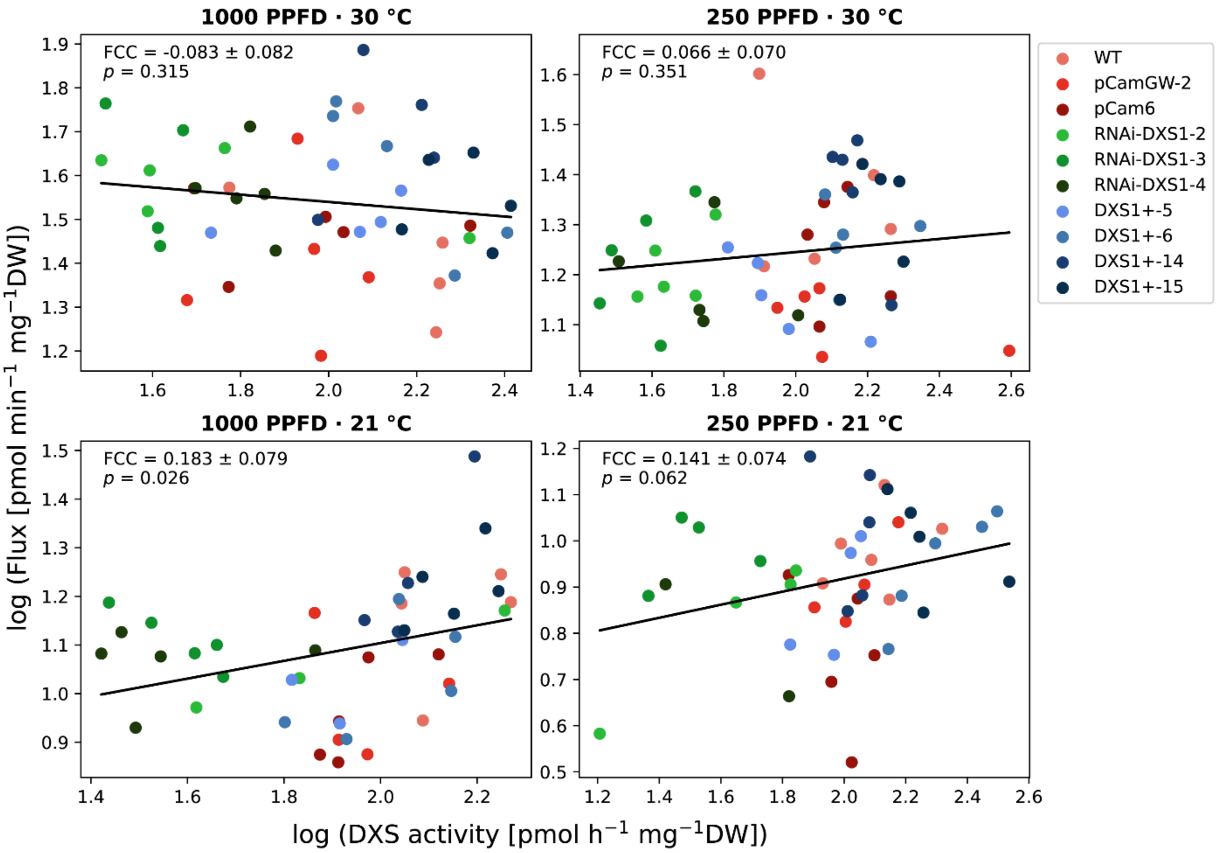

Supplemental Figure S3. Flux-control coefficients of grey poplar DXS for the MEP pathway under different conditions of light (1000 or 250 PPFD) and temperature (30 or 21ºC). Fluxes are plotted as a function of DXS activity in double-logarithmic space for the wild type (WT), empty vector controls (pCam), *DXS1*-silenced (RNAi-*DXS1*) and overexpression (*DXS1*+) lines. The FCCs were calculated from the slope of the linear regression for each environmental condition and are indicated on the graphs. The *p*-values refer to the respective linear regressions and indicate a significant difference in FCC ($p<0.05$) from a zero slope only for plants growing at 1000 PPFD and 21ºC.

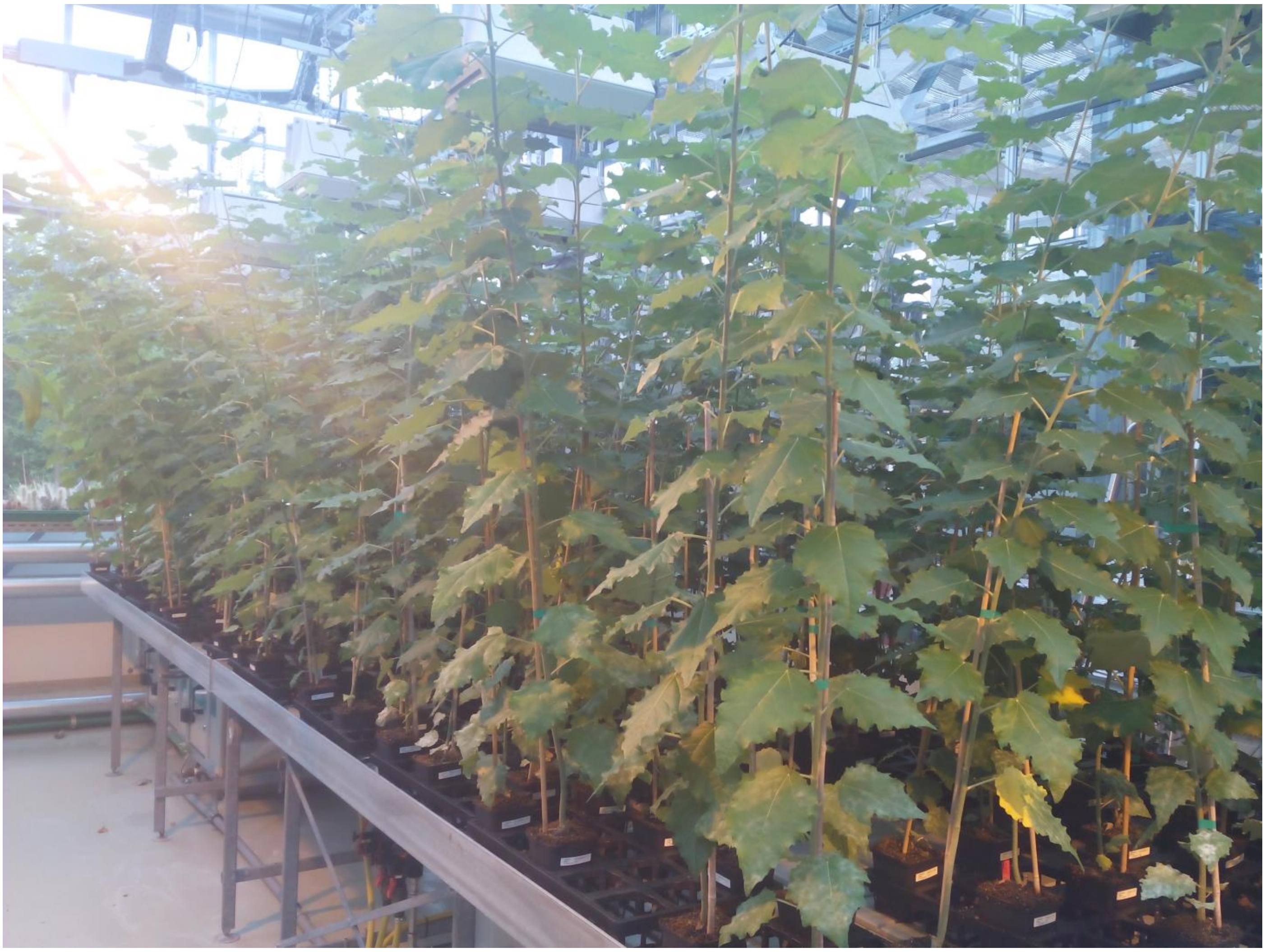

Supplemental Figure S4. *Populus x canescens* trees grown in the greenhouse at the Max Planck Institute for Chemical Ecology under the conditions described in the methods section.

Supplemental Table S1. Primer sequences and annealing temperatures used in the real-time PCR analysis

Gene name	Forward primer (5′→3′)	Forward primer (3′→5′)	T_{anneal}(°C)
PcDXS1	GGAAGAGCGAACAATGTGGTTG	GGTGGTGTTGGCCCATCTAAA	61
PcDXS1	AGGAACGAACAAGGTAGTCTCC	GTGGTATCGGCCCATCCAAG	62
PcDXR1	CTGGGGAGCAAGGAGTTGTA	TGTGAGCAAGAGGGAGAACA	51
PcDXR2	TCCTGGGGAGCAAGGAGTTATC	CGCAGCAACCGTAGGCTTTAG	59
PcCMK	GGCATTCCAATGGTTCTCATG	AAAGGCAGGAGGTTCCAAAT	51
PcHDR	CAAGTAACACCTCCCACCTTCA	CCCCATGATTCAACTTATAAGCTATTCTG	51
PcHMGR	GTGGGGCTACTAGTGTTTTGTTG	CGCAAACCTACTCGACCTGTTA	62
PcMVA	AATCATGGTTTGCTCCAATGC	CATTGGAATCCGCAAGACTCTA	60
PcActin	TGTCCGTGACATGAAGGAG	ACCAGGGAACATAGTGGAAC	54
